# Automated analysis of cardiovascular magnetic resonance myocardial native T_1_ mapping images using fully convolutional neural networks

**DOI:** 10.1186/s12968-018-0516-1

**Published:** 2019-01-14

**Authors:** Ahmed S. Fahmy, Hossam El-Rewaidy, Maryam Nezafat, Shiro Nakamori, Reza Nezafat

**Affiliations:** 1 Department of Medicine, Beth Israel Deaconess Medical Center and Harvard Medical School, 330 Brookline Ave, Boston, MA 02215 USA; 2 Biomedical Engineering Department, Cairo University, Cairo, Egypt

**Keywords:** T_1_ mapping, Convolutional neural networks, Automatic analysis, Myocardium segmentation

## Abstract

**Background:**

Cardiovascular magnetic resonance (CMR) myocardial native T_1_ mapping allows assessment of interstitial diffuse fibrosis. In this technique, the global and regional T_1_ are measured manually by drawing region of interest in motion-corrected T_1_ maps. The manual analysis contributes to an already lengthy CMR analysis workflow and impacts measurements reproducibility. In this study, we propose an automated method for combined myocardium segmentation, alignment, and T_1_ calculation for myocardial T_1_ mapping.

**Methods:**

A deep fully convolutional neural network (FCN) was used for myocardium segmentation in T_1_ weighted images. The segmented myocardium was then resampled on a polar grid, whose origin is located at the center-of-mass of the segmented myocardium. Myocardium T_1_ maps were reconstructed from the resampled T_1_ weighted images using curve fitting. The FCN was trained and tested using manually segmented images for 210 patients (5 slices, 11 inversion times per patient). An additional image dataset for 455 patients (5 slices and 11 inversion times per patient), analyzed by an expert reader using a semi-automatic tool, was used to validate the automatically calculated global and regional T_1_ values. Bland-Altman analysis, Pearson correlation coefficient, *r*, and the Dice similarity coefficient (DSC) were used to evaluate the performance of the FCN-based analysis on per-patient and per-slice basis. Inter-observer variability was assessed using intraclass correlation coefficient (ICC) of the T_1_ values calculated by the FCN-based automatic method and two readers.

**Results:**

The FCN achieved fast segmentation (< 0.3 s/image) with high DSC (0.85 ± 0.07). The automatically and manually calculated T_1_ values (1091 ± 59 ms and 1089 ± 59 ms, respectively) were highly correlated in per-patient (*r* = 0.82; *slope* = 1.01; *p* < 0.0001) and per-slice (*r* = 0.72; *slope* = 1.01; *p* < 0.0001) analyses. Bland-Altman analysis showed good agreement between the automated and manual measurements with 95% of measurements within the limits-of-agreement in both per-patient and per-slice analyses. The intraclass correllation of the T_1_ calculations by the automatic method vs reader 1 and reader 2 was respectively 0.86/0.56 and 0.74/0.49 in the per-patient/per-slice analyses, which were comparable to that between two expert readers (=0.72/0.58 in per-patient/per-slice analyses).

**Conclusion:**

The proposed FCN-based image processing platform allows fast and automatic analysis of myocardial native T_1_ mapping images mitigating the burden and observer-related variability of manual analysis.

**Electronic supplementary material:**

The online version of this article (10.1186/s12968-018-0516-1) contains supplementary material, which is available to authorized users.

## Introduction

Cardiovascular magnetic resonance (CMR) myocardial native T_1_ mapping [1–5] enables quantification of interstitial diffuse fibrosis [6] and has been increasingly used in diagnosis and prognosis of different cardiomyopathies [7, 8]. In myocardial T_1_ mapping, a set of T_1_ weighted images are acquired by changing the time between the preparation pulse and image acquisition [1–5] to generate different T_1_ weightings. The T_1_ value at each voxel is then estimated by fitting an exponential relaxation curve to the voxel intensities of the different T_1_ weighted images [4, 9]. This necessitates that voxels align perfectly on different images to avoid errors in estimation and increase the reproducibility [4]. Both respiratory and cardiac motion could cause artifact in T_1_ maps and should be addressed during acquisition or post-processing step. To minimize the impact of cardiac motion, T_1_ mapping is acquired during systolic or diastolic quiescent period within a short acquisition window [2, 10]. For respiratory motion, both breath-holding [1, 2] and free-breathing using slice tracking have been used [5, 11]. However, both techniques still require post-processing motion correction [12–14]. Numerous semi-automatic techniques are available to compensate this respiratory motion [12–14], however these methods are not effective in all patients [15].

T_1_ mapping analysis requires manual segmentation of T_1_ maps from different slices [14]. Endocardial and epicardial contours are drawn manually on the maps to delineate the myocardial areas. Regional T_1_ values (e.g. septal T_1_) can also be measured by drawing a region of interest (ROI) in the desired area. However an experienced reader is often needed for reproducible measurements [6, 16]. Despite the availability of semi-automatic and automatic techniques for cardiac cine [17–19] and flow [20, 21] imaging, there is no software for automatic analysis of myocardial tissue characterization images. Therefore, there is a need for automating the analysis of myocardial tissue characterization sequences such as T_1_ mapping.

Recent advances in deep learning technologies, namely convolutional neural networks, have shown potential for fully automated segmentation of the left [18, 19, 22–24] and right [24, 25] ventricles in cine and myocardial scarring in late gadolinium enhancement [26]. Deep convolutional neural networks comprises several layers of linear and nonlinear operations with millions of functional parameters [27, 28]. The large number of network parameters allows representation of objects with diverse appearance and shape patterns. Deep learning based myocardium segmentation pipelines usually employs a single convolutional neural network architecture [24, 26]. However, a cascade of different neural network architectures has been also used to achieve different tasks such as locating the heart within the imaging field of view and extracting the myocardium boundaries [22, 25]. Also, combining classical image processing methods (e.g. level sets and deformable models) with deep learning has been proposed to refine the segmentation results [19, 23].

In this study, we propose to develop and evaluate a fully automated analysis platform for myocardial T_1_ mapping using fully convolutional neural networks (FCN) [27, 28]. The proposed method automates the analysis of short-axis T_1_ weighted images to estimate the myocardium T_1_ values. The performance of the proposed approach was evaluated against manual T_1_ calculation.

## Methods

**Fig. 1:**
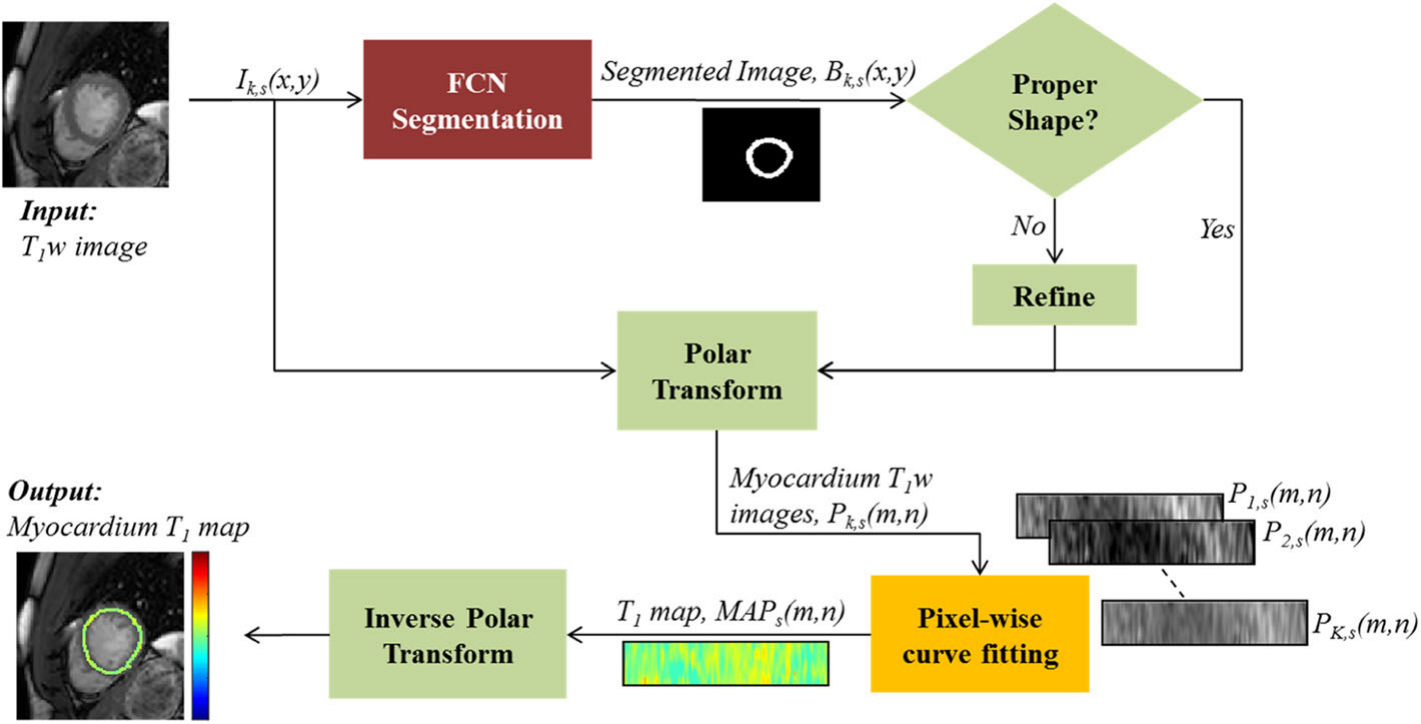
Pipeline for myocardium T_1_ map reconstruction. The myocardium in an input T_1_ weighted (T_1_w) image is first segmented using a fully convolutional neural network (FCN). The segmented myocardium is refined if needed (see text for details) and transformed into polar coordinates. All T_1_w images at a given slice are used to estimate the myocardium T_1_ map, which is displayed after applying inverse polar transformation

The proposed workflow for automated T_1_ map analysis is summarized in Fig. 1. In summary, the first step includes FCN-based myocardium segmentation, with additional automatic evaluation and refinement of the segmented myocardium shapes. The second step includes transformation of the segmented myocardium within the different T_1_ weighted images onto a polar coordinate system, which implicitly aligns the segmented myocardium regions. The myocardium T_1_ maps are then estimated (in the polar coordinate system) and transformed to the Cartesian coordinates for conventional map visualization. Validation of the proposed method was accomplished by comparing the automatically calculated myocardium T_1_ values to a current state-of-the-art semi-automatic T_1_ mapping technique [13]. Manual analysis by two independent readers was used to assess the inter-observer variability. Both per-slice and per-patient analyses were performed for all validation experiments. The following subsections provide further insight into the steps.

### Myocardium segmentation

#### Fully convolutional neural networks

**Fig. 2:**
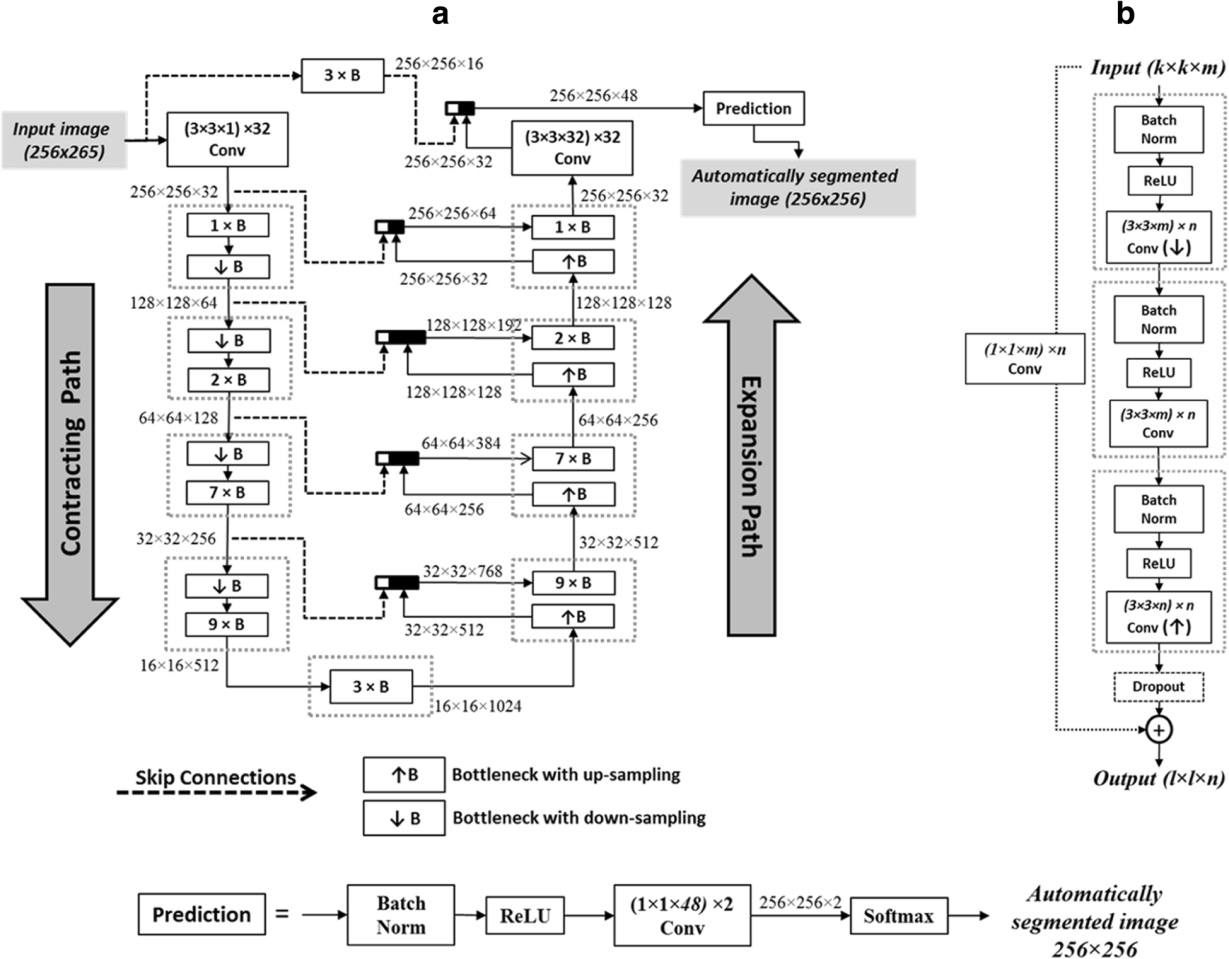
The fully convolutional neural network architecture (**a**) comprises a number of building blocks, referred to as bottlenecks (**b**). An input 256 × 256 image undergoes a series of convolutions (Conv), nonlinear rectifications (ReLU), and batch normalizations (Norm). Down-sampling (↓) and up-sampling (↑) of the processed images are applied in the contracting and expansion paths, respectively. The *l, k, m,* and *n* values in (**b**) are determined by the image size and number of channels at the input and output of each bottleneck as shown in (**a**)

A deep FCN based on the U-Net architecture [29] was used for myocardium segmentation (Fig. 2). FCN is a special class of neural networks where all the network layers are based on convolutional sub-layers [29]. The FCN input is a two-dimensional 256 × 256 T_1_ weighted image, *I*_*k,s*_(*x,y*), acquired at slice *s* (= 1 to 5) and inversion time TI_*k*_ (with *k* = 1 to 11), and the output is a binary image, *B*_*k,s*_(*x,y*), of the same size and with pixels labeled as myocardium or background. Our network comprised 149 processing layers with a total of approximately 9 million kernels. The basic structural unit in U-Net, referred to as a bottleneck (Fig. 2)b, contains three functional layers: batch-normalization which accelerates the network training [2, 30]; a rectified linear unit (ReLU) which introduces the nonlinearities required to model complex operations involved in the image segmentation; and [3] spatial convolution with a set of *n* kernels of size *s × s × w*, where the values of *s* and *w* are as indicated in Fig. 2. The weights of the convolutional kernels are the FCN parameters that are estimated during the training process. Spatial down-sampling (or up-sampling) operations are also applied during convolution and are combined with doubling (or halving) the number of kernels, *n*. To prevent overfitting, a dropout layer is used in each bottleneck to randomly (with 50% probability) pass or block the processed data [31]. A cross-entropy loss function was used to represent the network error and an Adam optimizer was used to estimate the network parameters [32]. A weight decay of 0.001 was used for regularization [33]. The final stage in the FCN network is a prediction block that generates two probability maps representing the likelihood of each pixel to belong to a background or a myocardium region. A softmax layer is then used to produce a binary image with pixels assigned 1 or 0 for myocardium or background, respectively.

#### Post processing and automated segmentation assessment

The binary image resulting from FCN segmentation was enhanced through a set of post-processing operators. First, an area-filter was applied to remove all segmented objects with an area less than 5 cm^2^ and maintaining only the largest segmented object. The segmented myocardium was then automatically assessed for potential shape errors; e.g. absence of an annulus shape. A proper myocardium shape was quantified by two geometric parameters: Euler number and Eccentricity. The Euler number represents the number of holes in the object and should equal zero for the typical annulus shape of the myocardium in short axis slices. The Eccentricity represents deviation from a perfect circle (= zero for a circle and = 1 for a line segment). The training dataset was analyzed to determine the typical range of myocardium eccentricity and the maximum was 0.65. If the segmented myocardium has Euler number ≠0 or eccentricity > 0.65, it is marked as an improper segmentation and becomes eligible for automatic shape refinement.

#### Automatic segmentation refinement

Given an image, *I*_*k,s*_(*x,y*), and its segmentation, *B*_*k,s*_(*x,y*), that was identified to have a segmentation shape error, the binary image, *B*_∞, *s*_(*x,y*), resulting from segmenting the image with the longest inversion time was used to refine *B*_*k,s*_(*x,y*). The image with the longest inversion time was chosen due to its high myocardium-to-blood contrast which leads to increased segmentation reliability. The refinement was done by applying an affine transformation (translation, rotation and scaling) to the binary image *B*_∞, *s*_(*x,y*) to obtain a refined binary image *B̃*_*k,s*_(*x,y*) with maximum overlap with *B*_*k,s*_(*x,y*). It is worth noting that if *B*_∞, *s*_(*x,y*) was found to have segmentation error, no refinement was done and the image *B*_*k,s*_(*x,y*) was excluded from analysis. An image segmentation is considered successful if a myocardium with a valid shape is produced (whether automatic refinement was applied or not).

### T_1_ map reconstruction and analysis

To align the myocardium regions in different T_1_ weighted images, the segmented myocardium in a given image, *I*_*k,s*_(*x,y*), was transformed to polar coordinates on a uniform grid (Additional file 1: Figure S1). The origin of the polar coordinates was located at the center-of-mass of the segmented myocardium. The transformation was achieved by sampling the myocardium intensities along 360 radial rays, with angular spacing of 1^o^, from the origin to the epicardium. A number of *C* intensity values were sampled between the endocardium and the epicardium along each ray. The result was a rectangular image, *P*_*k,s*_(*m,n*), of size *C* × 360, which represents a temporary image in the polar coordinates, where the T_1_ map is generated and then inverse transformed to the Cartesian coordinates. To avoid loss of data during transformation; i.e. avoid many-to-one transformation, *C* was arbitrary fixed to a value (= 20) that is larger than the maximum myocardium thickness found in the training set (= 15 pixels). The set of all transformed images at a given slice location, *P*_*k,s*_(*m,n*) for all inversion times *k* (= 1 to 11), was then used to estimate the myocardium T_1_ map, *MAP*_*s*_(*m,n*), at the given slice. This was achieved by performing pixel-wise curve fitting of a 2-parameter model to the myocardium intensities, *P*_*k,s*_ (*m,n*) for all *k* values [5]. T_1_ map reconstruction was done only for slices with at least 8 successfully segmented T_1_ weighted images. Finally, the resulting 20 × 360 T_1_ map was inverse transformed to the Cartesian coordinates. While any of the T_1_ weighted images could be used as a reference for the inverse polar transformation, the image with shortest inversion time was used to match the reference of the semi-automatic method. Inverse polar transformation is accomplished by determining the polar coordinate of each myocardium point on the Cartesian grid of the reference image and estimating its T_1_ value using bilinear interpolation of the reconstructed polar T_1_ map.

The pixels in sub-endo and sub-epicardial borders were excluded in automatic measurements to mimic the manual analysis. This was accomplished by automatically pruning the segmented myocardium, where the myocardium skeleton (i.e., central contour of 1 pixel width) [34] was extracted and dilated (using image morphological operator) to one-third of the segmented myocardium mean wall thickness. The thickness was chosen arbitrary and could be changed manually.

The global and regional myocardium T_1_ values were calculated by averaging the T_1_ values in the reconstructed maps over all 5 slices and over each slice, respectively. Any pixel with a T_1_ value outside the acceptance range for native T_1_ at 1.5 T (i.e. 850 ms to 1500 ms) was excluded from the average T_1_ calculations.

### Image acquisition

We prospectively recruited 665 consecutive patients (526 male; age 56 ± 15 years) with known or suspected cardiovascular diseases referred for a clinical CMR exam during the period from 2014 to 2017. All patients provided consent at the time of examination for use of their imaging data in research; the imaging protocol was approved by the Institutional Review Board. Patient data was handled in compliance with the Health Insurance Portability and Accountability Act. Imaging was performed using a 1.5 T Philips Achieva system (Philips Healthcare, Best, The Netherlands) with a 32-channel cardiac coil. The imaging protocol included free-breathing, respiratory-navigated, slice-interleaved T_1_ (STONE) sequence [5] with the following parameters: TR/TE = 2.7/1.37 ms, FOV = 360 × 351 mm^2^, acquisition matrix = 172 × 166, voxel size = 2.1 × 2.1 mm^2^, linear ordering, SENSE factor = 1.5, slice thickness = 8 mm, bandwidth = 1845 Hz/pixel, diastolic imaging, and flip angle = 70^o^. Each patient imaging set was comprised of 55 images representing a stack of five short axial slices covering the left ventricle (LV) from base to apex. At each slice location, eleven T_1_ weighted images were acquired at eleven different inversion times, TI, (= ∞, 115 ms, 115 ms + RR, 115 ms + 2 RR, …, 115 ms + 4 RR, 350 ms, 350 ms + RR, …, 350 ms + 4 RR; and RR is duration of the cardiac cycle) [5]. The matrix size of all images was unified to 256 × 256.

The image dataset was split into two subsets for: 1) FCN training and testing and 2) validation of the T_1_ calculations. The first dataset contained 210 patients (134 male; 57 ± 14 years; total of 11,550 T_1_ weighted images) and was used to train and test the proposed FCN network. The myocardium of the LV in each image was manually segmented (HE, 4 year experience in medical image analysis); the resulting binary image was used as the segmentation reference standard. The dataset was then split at random into training and testing subsets containing 63 patients (total of 3465 images) and 147 patients (total of 8085 images), respectively.

The second image subset contained 455 patients (392 male; 56 ± 15 years) and was used to assess the agreement between T_1_ values computed by the automated versus the manual analysis. An experienced reader (SN, with 8 year CMR experience) used an in-house T_1_ map reconstruction tool to estimate T_1_ values for each myocardium slice [13]. First, for each slice, the reader manually delineated the endocardium on a reference T_1_ weighted image. Then, intensity-based similarity metrics were used to estimate the global LV motion of the T_1_ weighted images relative to the reference T_1_ weighted image. A regularized optical flow based method was then used to refine the image registration of the registered T_1_ weighted images to the reference using an optical flow based algorithm [13]. The resulting T_1_ maps were then manually processed to select a ROI within the myocardium that excluded all areas suspected of imaging or mapping artifacts. To assess the inter-observer variability, a subset of 40 patients (24 male; age 56 ± 11.7 years) was selected at random and manually processed by a second reader (MN, with 5 year CMR experience) to reconstruct and analyze the T_1_ maps as described above.

### Implementation and evaluation

Network training was performed for 48 h (number of iterations = 6700) using a manually annotated dataset described below. The intensity dynamic range of each image was normalized by subtracting the mean and dividing by the standard deviation (SD) of the image pixel intensities. A transfer learning approach was employed to speed up training and to mitigate the requirement of large training datasets [35, 36]. That is, instead of random initialization of the network parameters, we re-used the optimal parameter values of a previously trained FCN. The re-used FCN had the same architecture as the current network and was trained (using 6305 images from 831 patients) to segment the myocardium in late gadolinium enhancement CMR images [26]. Image augmentation was also used to reduce overfitting [37], where each training image pair (T_1_ weighted image and its corresponding manually segmented image) was used to synthesize a number of training image pairs. Several methods of image augmentation were presented in literature, where geometric and/or intensity transformations can be used to synthetize the training images [38]. In our network, no intensity transformation was used for image augmentation because of the naturally high dynamic range of the image intensities and contrast in the T_1_ weighted images. Geometric image transformation was used through random translation, mirroring and elastic deformation of the training images with probabilities of 0.95, 0.95, 0.5, respectively. The FCN segmentation error was measured as by a cross-entropy loss function (between the FCN output and the manual segmentation). For network parameter estimation, the loss function was optimized using the Adam method with a learning rate of 0.001 and exponential decay rate [32].

The performance of the FCN network for myocardial segmentation was evaluated using the independent testing images. Dice similarity coefficient (DSC) was used to measure the overlap between the automatically and manually segmented myocardium in each testing image. The DSC ranges from 0 to 1 with higher values indicating higher similarity in shape between the automatically and manually segmented regions [39].

Network training and testing was performed on an Intel Core i7-6700 K CPU workstation with NVIDIA GeForce GTX Titan 12GB GPU. The network was implemented using Python (Python Software Foundation, Wilmington, Delaware, USA) with Tensorflow machine learning framework (Google Inc., California, USA).

### Data analysis

Calculated T_1_ values were expressed as mean ± SD per patient and per slice. The performance of the automatic T_1_ analysis was evaluated by analyzing the agreement between the automated and manual T_1_ calculations. The Pearson correlation coefficient, *r*, was used to examine the linear relationship (with zero intercept) between the automated and manual T_1_ calculations. The Bland-Altman analysis was also used to assess the biases and limits of agreement between automated and manual T_1_ calculations.

Intraclass correlation coefficient (ICC) was used to assess inter-observer agreement. Inter-observer agreement was assessed between each pair of observers: automatic vs reader 1, automatic vs reader 2, and reader 2 vs reader 1. Intra-observer variability of the presented mapping and analysis method is deterministically zero (due to full automation) and thus was not studied using a dedicated experiment. All analyses were done on a per-patient and per-slice basis. All statistical analyses were performed using the statistical toolbox of Matlab (Mathworks Inc., Natick, Massachusetts, USA).

## Results

**Fig. 3:**
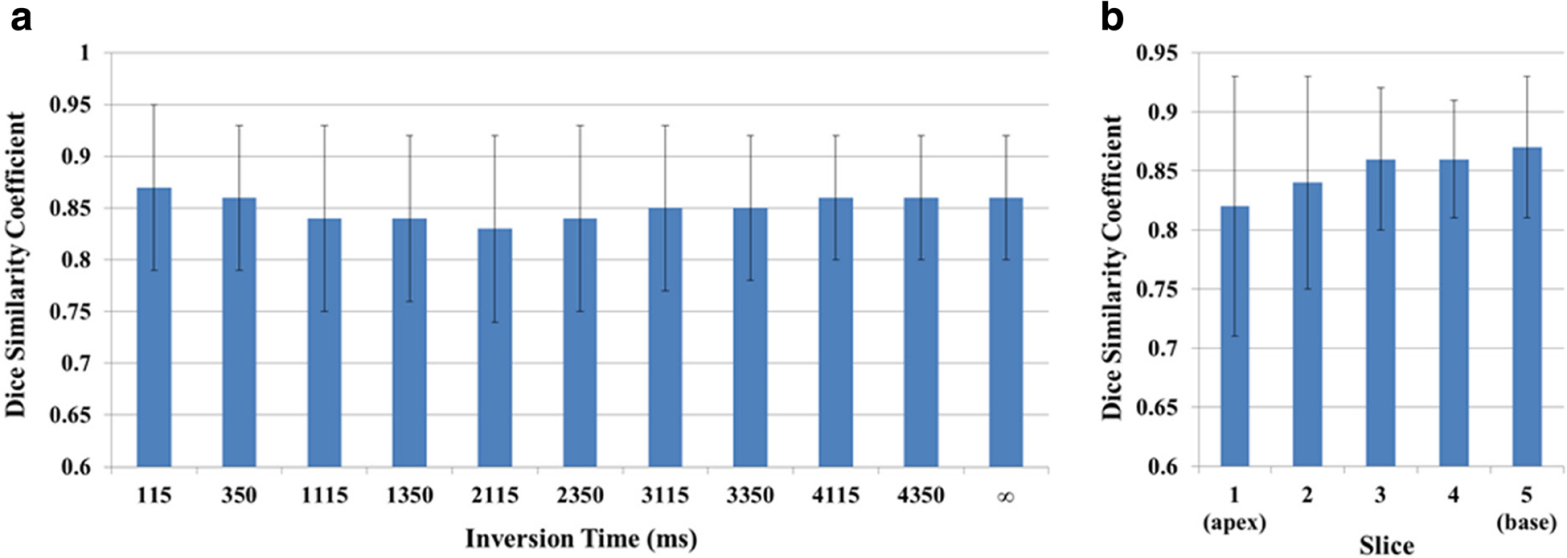
The Dice similarity coefficient of the automatic segmentation averaged over 147 patients (7382 images) categorized by inversion time (**a**) and slice location (**b**). Error bars represent standard deviation

**Fig. 4:**
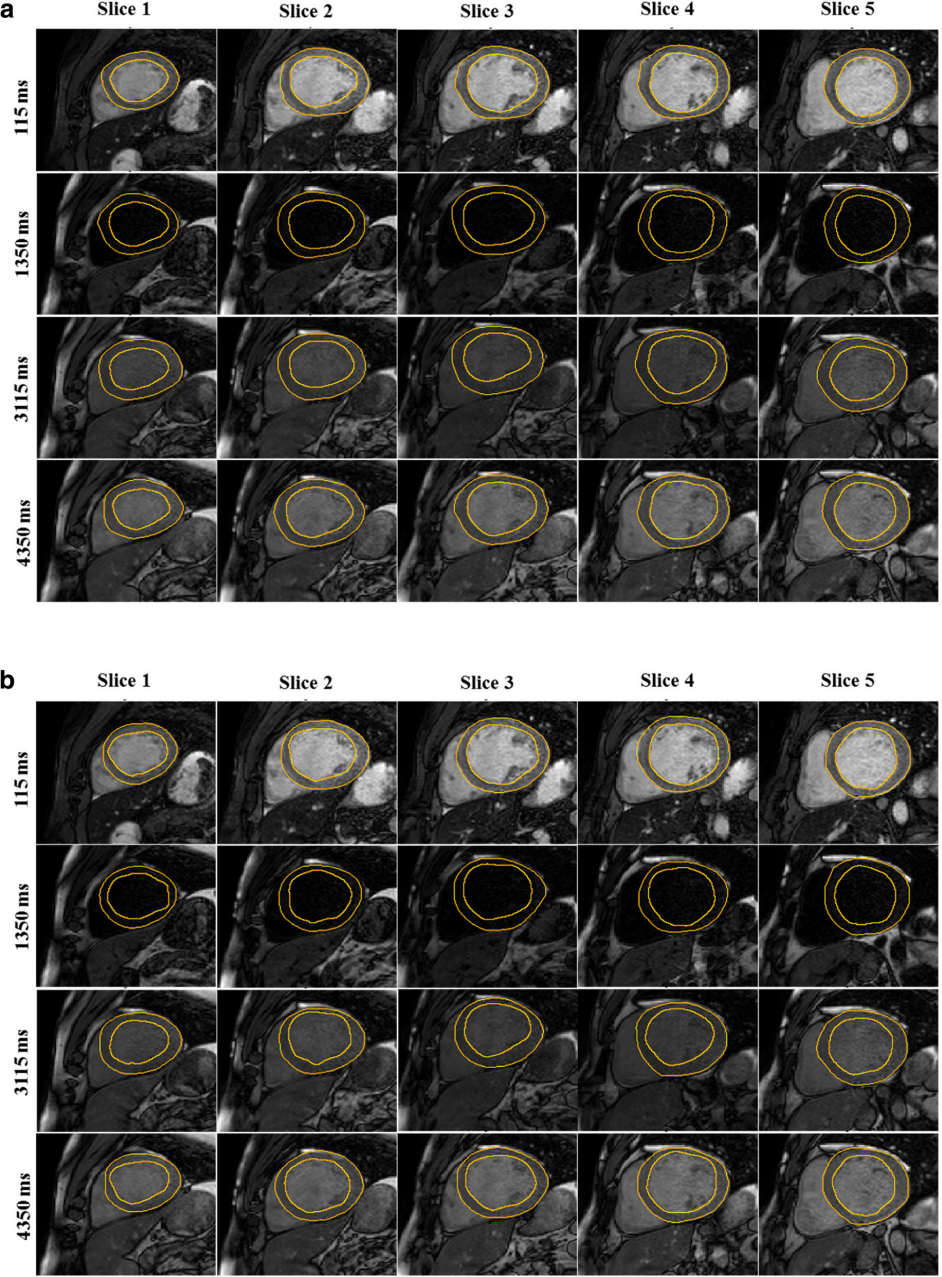
Automatic (**a**) and the corresponding manual (**b**) segmentation of T_1_ weighted images for five slices (columns) and four different inversion times (rows) for one patient

**Fig. 5:**
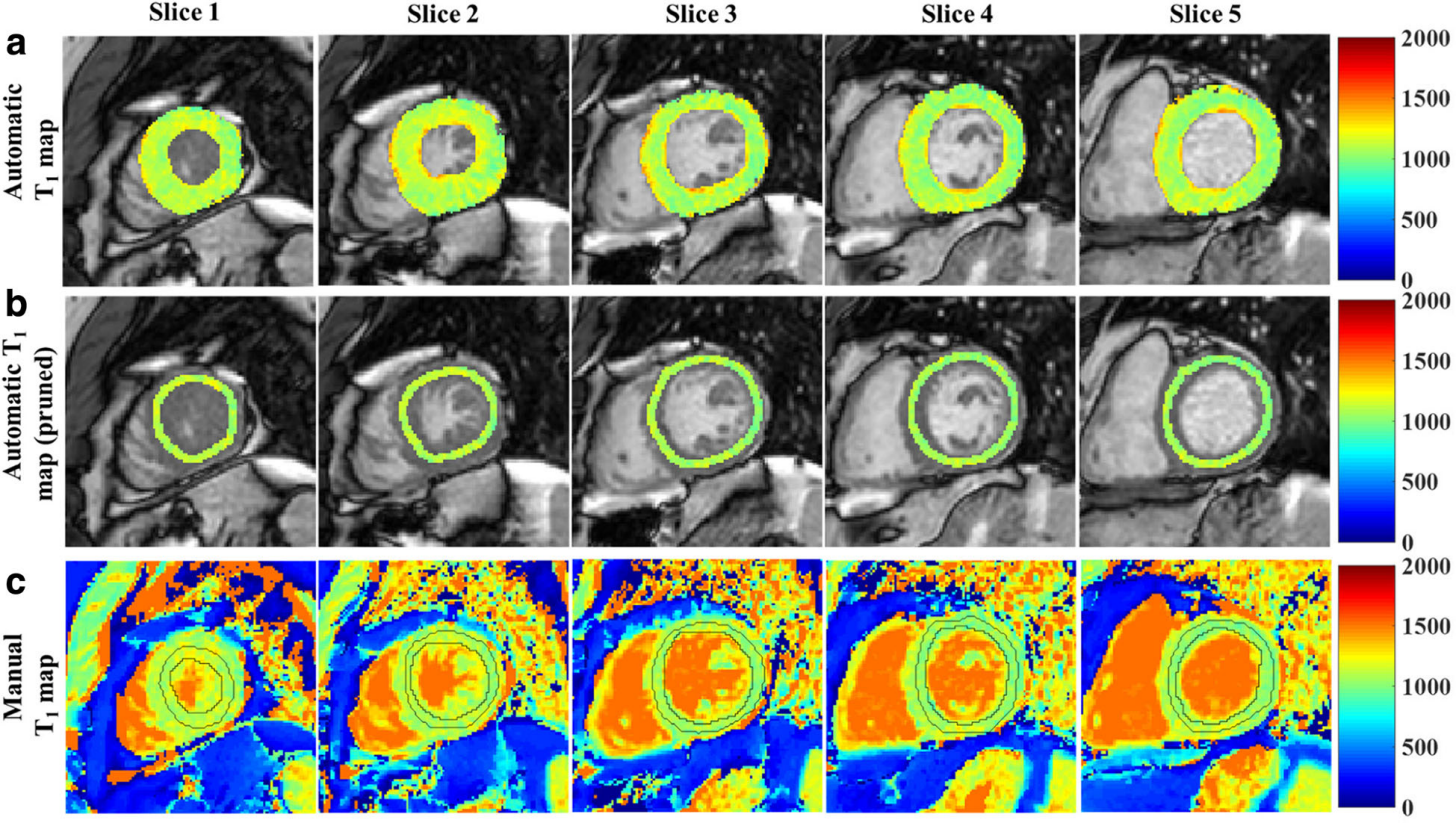
Myocardial T_1_ mapping at five short axial slices (apex to base from left to right respectively) of the left ventricle of one patient. Automatically reconstructed map before (**a**) and after (**b**) pruning overlaid on a T_1_ weighted image with shortest inversion time; (**c**) Manually reconstructed T_1_ map. The contours in (**c**) represent the myocardium region of interest manually selected by the reader

**Fig. 6:**
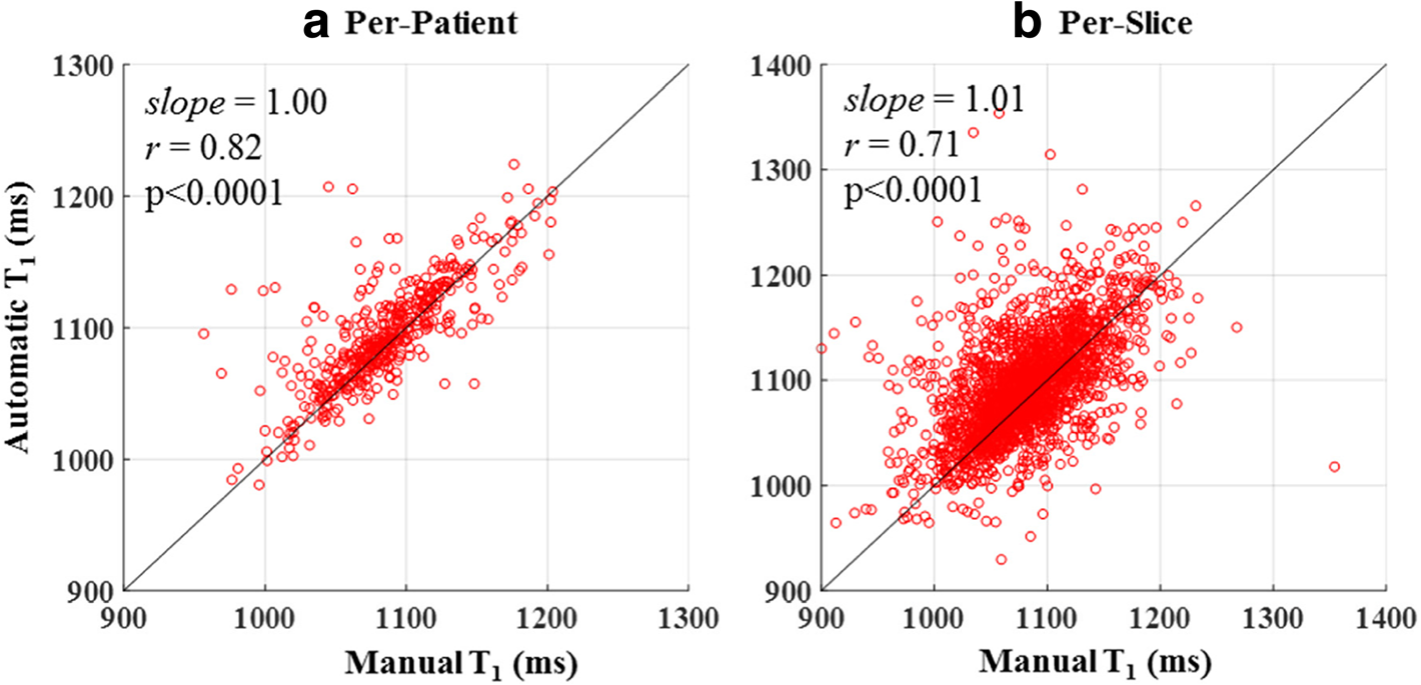
Scatter plots of the automatic versus manual myocardium T_1_ values averaged over the patient volume (**a**) and each imaging slice (**b**). Solid lines represent the unity slope line

**Fig. 7:**
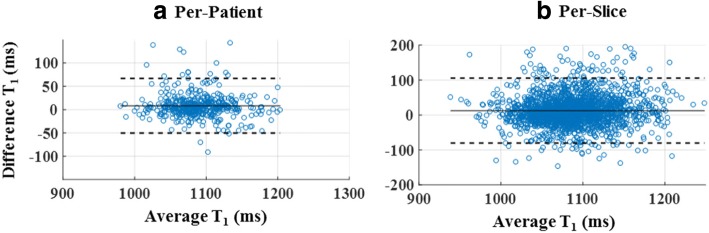
Bland-Altman plots of the automatic versus manual myocardium T_1_ values averaged over the patient volume (**a**) and each imaging slice (**b**). Solid and dashed lines represent the bias and ± 2SD limits, respectively

The FCN successfully segmented the myocardium in 7382 testing images (91.3% of 8085 images) with an overall DSC score of 0.85 ± 0.07 (Fig. 3) after applying refinements. The computation time for segmenting a single T_1_ weighted image was less than 0.3 s. Automatic refinement of the myocardium segmentation was done to 241 images (3% of 8085 images) (Additional files 2: Figure S2 and Additional file 3: Figure S3). Table 1 summarizes the number of slices with correct, failed or refined segmentation. The FCN segmentation of the myocardium showed good overlapping with the manually segmented myocardium with mean DSC greater than 0.82 in all slices at all inversion times (Figs. 3 and 4). In the mapping validation images (2275 T_1_ maps for 455 patients), automatic reconstruction of T_1_ maps was successful in 1982 slices (87.1% of 2275 slices) in 449 patients (98.7% of 455 patients). The success rate of map reconstruction in the non-apical slices (1682 slices; 92.4% of 1820 slices) was higher than that in the apical slices (300 slices; 65.9% of 455 slices). The automatically and manually calculated T_1_ values within the myocardium ROI (Fig. 5) averaged over all patients were 1091 ± 59 ms and 1089 ± 59 ms, respectively. The automatically reconstructed T_1_ maps showed a strong correlation with the manually reconstructed T_1_ values in per-patient (*r* = 0.82; *slope* = 1.01; *p* < 0.0001; 449 patients) (Fig. 6)a and per-slice (*r* = 0.74; *slope* = 1.01; *p* < 0.0001; 2275 slices) analyses (Fig. 6) b. The correlation between the automatic and manual T_1_ mean values were comparable across the five slice locations (*r/slope* = 0.74/1.03, 0.76/1.02, 0.73/1.0, 0.76/1.01, and 0.75/1.01 for the 5 slices from apex to base respectively; *p* < 0.0001 for all slice locations). The automated and manual T_1_ calculations were in good agreement with 95% of the measurements located between the limits-of-agreement lines in per-patient (9.6 ± 86.6 ms) and per-slice (12.9 ± 110.1 ms) analyses (Fig. 7). The automated T_1_ calculations showed good agreement with the manual calculations in the per-patient (ICC = 0.86 and 0.74 for automatic vs. reader 1 and reader 2, respectively) and per-slice (ICC = 0. 56 and 0.49 for automatic vs. reader 1 and reader 2, respectively) analyses (Table 2). The ICC between the two expert readers was 0.72 and 0.58 in the per-patient and per-slice analyses, respectively. The average computation time for generating a T_1_ map of one slice was less than 15 s (segmentation and refinement = 5 s, polar transformation = 7.5 s, curve fitting = 1.5 s).

**Table 1:** The number of images with correct, failed or refined segmentation reported for 147 patients (total of 7382 images) and categorized by the slice location and inversion time (TI)

*TI (ms)* ^a^		115	350	1115	1350	2115	2350	3115	3350	4115	4350	∞
Number of Images	*Correct*	597	649	637	634	643	654	664	662	665	668	668
	*Failed*	82	59	61	63	65	61	61	61	61	62	67
	*Refined*	56	27	37	38	27	20	10	12	9	5	0
Slice location			1 (apical)		2		3		4		5 (basal)	
Number of Images	*Correct*		1093		1393		1533		1560		1562	
	*Failed*		432		165		43		38		25	
	*Refined*		92		59		41		19		30	

^a^Assuming cardiac cycle duration of 1000 ms

**Table 2:** Inter-observer analysis of the automated and manually calculated myocardium T_1_ maps in per-patient and per-slice analyses

	Per-patient	Per-slice
Automatic vs Reader 1	ICC = 0.86; CI = 0.74–0.92	ICC = 0.56; CI = 0.44–0.67
Automatic vs Reader 2	ICC = 0.74; CI = 0.55–0.86	ICC = 0.49; CI = 0.36–0.61
Reader 2 vs Reader 1	ICC = 0.72; CI = 0.53–0.84	ICC = 0.58; CI = 0.48–0.67

*ICC* intraclass correlation coefficient; CI = 95% confidence interval

## Discussion

In this work, we introduced an automated method for combined segmentation, alignment, and T_1_ estimation of the myocardium. Myocardium segmentation was achieved using a deep learning approach where a FCN was used to segment the myocardium in all slice locations and all inversion times. Typically, multiple shape and appearance models are needed to capture the wide variation of myocardium shapes and intensity patterns in different T_1_ weighted images [14]. However, despite highly variable shapes and intensity patterns in the images, the employed FCN showed good performance as assessed by DSC. The developed FCN-based analysis platform showed the potential to mitigate the requirement of tedious manual T_1_ mapping analysis, with good agreement between automatic and manual calculations. Furthermore, inter-observer variability of the automatic vs manual calculations was comparable to that between the two expert readers.

In this work, we employed the same deep neural network architecture (Unet) that we previously trained and used to segment the myocardium and scar in short-axis late gadolinium enhancement (LGE) images [26]. We also used the estimated network weights of the previously trained network to initialize the weights of our network. Deep neural network architectures requires a large training set to allow generalization of the trained model through seeing all potential variations of the images [36]. In our study, limited size of the training image sets was supported by employing two standard techniques for improving network training; namely, data augmentation and transfer learning [35, 36]. Data augmentation has been shown previously to be effective to reduce over-fitting and thus boost the segmentation performance in images from outside the training set [40, 41]. Also, transfer learning approach was shown to outperform training-from-scratch approach (i.e. initialization of network parameters using random values) [42]. Networks trained using transfer learning approach were also shown to be more robust to the size of training sets compared to networks trained from scratch [42]. Several techniques based on deep learning have been proposed and evaluated for the segmentation of cine images [18, 19, 22–24, 43–45]. Network design varied among the different methods and included using only one fully convolutional neural network [24, 44], multi-stage convolutional neural networks [45], or cascaded convolutional and auto-encoder networks [25]. Convolutional neural networks have been also combined with classical image processing techniques such as deformable models [19] and level sets [23] aiming to refine the segmentation results. These methods achieved high segmentation accuracy of the LV cavity (DSC =0.9–0.94) but determining the parameters of the refinement algorithm can be a limitation. Oktay et al. showed that applying anatomical shape constraints to the convolutional neural networks can improve the segmentation accuracy without a need for a refinement step [43]. A non-convolutional neural network model was also proposed for cine image segmentation, where the segmentation problem was formulated as parameter regression rather than conventional pixel classification. In this formulation, the network was trained to estimate the radial distance between the myocardium boundary points and the myocardium centroid [18]. Any of these methods can be readily incorporated into our analysis framework. However, further investigation is warranted to adapt these methods to segment T_1_ weighted images and evaluate T_1_ map analysis.

Unlike current T_1_ mapping analysis methods that require explicit image registration of the T_1_ weighted images prior to T_1_ map reconstruction [12–14], our method inherently aligns the myocardium regions through polar transformation. For example, maintaining the location of the origin point of the polar coordinates at the center-of-mass of the segmented myocardium results in inherent correction of the global translational heart motion [46]. Also, resampling of the segmented myocardium via a uniform polar grid results in non-rigid alignment of the myocardium across all T_1_ weighted images. Utilization of geometric transformations leads to image alignment that is independent of the image intensity and contrast, and thus overcomes a limitation of conventional intensity-based image registration methods [12–14]. The proposed workflow did not include an explicit motion correction and instead relied on polar transformation and alignment to compensate for motion. An alternative approach is to apply motion correction to the T_1_ weighted images, reconstruct the T_1_ maps, and then use deep-learning based segmentation of myocardium from T_1_ maps. While this approach can be simpler, it might be limited by cascading the errors of the motion correction and the segmentation steps. Also, training the network to segment the myocardium in presence of residual motion artifacts can be challenging. A dedicated study is needed to investigate the performance of this workflow.

The polar grids have been previously used to register the myocardial strain and displacement maps using ultrasound imaging [47, 48]. The myocardium contours were first extracted at each cardiac time frame by means of semi-automatic tracking and then a polar grid was used to accumulate the displacement values of the deforming myocardium. One limitation of our method is that inaccurate myocardium segmentation can lead to erroneous T_1_ maps especially at the boundaries. However, T_1_ mapping errors at the myocardium boundaries are common to T_1_ mapping techniques due to partial volume effects and/or residual uncompensated motion effects, which necessitate manual exclusion of erroneous regions. In our method, these errors can be reduced by automatic pruning of the segmented myocardium.

The automatic refinement used in this work is a simple form of affine binary image registration, where the best segmentation mask is aligned with any given mask at the same slice location that has improper myocardium shape. An additional advantage of this approach is the efficient computations that result from confining image alignment and curve fitting to the myocardium regions-of-interest, rather than the entire field-of-view [13, 14].

The automated T_1_ calculations showed strong agreement with manual calculations in both per-patient and per-slice comparisons. Residual biases in automated T_1_ calculations might not necessarily correspond to T_1_ estimation errors and may be due to the inherent differences between the two methods of reconstructing and analyzing the T_1_ maps. In 12.9% of the slices, the myocardium was detected in less than eight T_1_ weighted images, which we set as the minimum number of T_1_ weighted images per slice required for T_1_ map reconstruction. These slices can be processed using manual or semi-automatic analysis. Alternatively, reconstruction might be allowed using fewer T_1_ weighted images to increase the success rate but might impact the accuracy of the T_1_ calculations. The failed reconstruction cases were mostly apical slices where the success rate (66%) was lower compared non-apical slices (92%). This is directly related to the higher segmentation failure of the myocardium at the apical slices, which is commonly encountered in myocardial segmentation techniques due to the blurred myocardium boundaries caused by motion artifacts or partial voluming [49, 50].

In our study, we used STONE sequence for T_1_ mapping, which results in 11 T_1_ weighted images spanning a relatively high dynamic range (due to the use of inversion recovery pulses). Training the FCN with images of diverse contrast, combined with data augmentation, and allowed a higher level of abstraction in learning the important image features. The roughly similar image contrast and dynamic range between STONE and other inversion recovery based techniques warrants validation of extending our trained FCN-based method to automate T_1_ map analysis in other mapping sequences such as modified Look-Locker inversion recovery (MOLLI) and [1] and shortened modified Look-Locker inversion recovery (ShMOLLI) [2]. Extension of our trained FCN to segment saturation-recovery based sequences such as saturation recovery single-shot acquisition (SASHA) [3], or combined inversion-recovery and saturation-recovery such as SAPPHIRE [4], are yet to be studied to investigate its reliability for analyzing T_1_ weighted images with inherent elevated noise levels. The additional post-processing of the FCN output was needed to correct for improper automatic segmentation of the LV structure. Alternative training strategies, including training of a separate network for each T_1_ weighted image, may be useful to improve the FCN performance and avoid heuristic post-processing. One limitation of this study is the lack of a ground truth for the myocardial T_1_ maps. Also, we did not investigate the capacity of the proposed analysis method to automate post-contrast T_1_ mapping and extracellular volume (ECV) mapping.

## Conclusion

The proposed FCN-based image processing platform allows fast and automatic analysis of myocardial native T_1_ mapping images mitigating the burden and observer-related variability of manual analysis.

## Additional files

Additional file 1: **Figure S1.** Transformation of the segmented myocardium into a uniform grid of size 20 × 360 in the polar coordinates. The origin of the polar coordinates is located at the center of mass of the segmented myocardium. (DOCX 115 kb)

Additional file 2: **Figure S2.** Example results of the automatic segmentation before and after refinement. (DOCX 470 kb)

Additional file 3: **Figure S3.** Effect of area filter on the output of the neural network. (a) input T1 weighted image; (b,c) network output before and after area filtering, respectively; (d) manual segmentation. (DOCX 225 kb)

## Abbreviations

CI: Confidence interval
CMR: Cardiovascular magnetic resonance
DSC: Dice similarity coefficient
ECV: Extracellular volume
FCN: Fully convolutional neural network
GRE: Gradient recalled echo
ICC: Intraclass correlation coefficient
LV: Left ventricle
MOLLI: Modified Lock-Looker inversion recovery
ROI: Region of Interest
SAPPHIRE: Saturation pulse prepared heart rate independent inversion-recovery
SASHA: Saturation recovery single-shot acquisition
SD: Standard deviation
ShMOLLI: Shortened modified Lock-Looker inversion recovery
SNR: Signal to noise ratio
STONE: Slice-interleaved T_1_
TE: Time of echo
TI: Inversion time
TR: Time of repetition
