# Supplementary material for: Automated analysis of cardiovascular magnetic resonance myocardial native T1 mapping images using fully convolutional neural networks
Source: J Cardiovasc Magn Reson. 2019 Jan 14;21:7. doi: 10.1186/s12968-018-0516-1 (PMC6330747; doi:10.1186/s12968-018-0516-1)
Supplement: Supplementary file 1 — Figure S1. Transformation of the segmented myocardium into a uniform grid of size 20 × 360 in the polar coordinates. The origin of the polar coordinates is located at the center of mass of the segmented myocardium. (DOCX 115 kb) [file 12968_2018_516_MOESM1_ESM.docx]

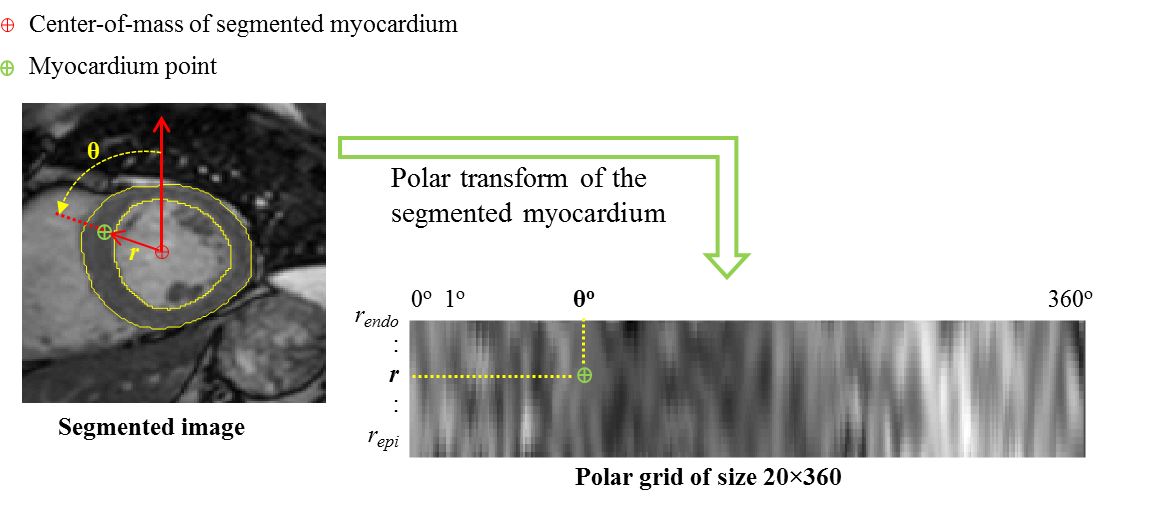


**Figure S1**. Transformation of the segmented myocardium into a uniform grid of size 20x360 in the polar coordinates. The origin of the polar coordinates is located at the center of mass of the segmented myocardium.
