# Supplementary figures and images for: Automated analysis of cardiovascular magnetic resonance myocardial native T1 mapping images using fully convolutional neural networks
Source: J Cardiovasc Magn Reson. 2019 Jan 14;21:7. doi: 10.1186/s12968-018-0516-1 (PMC6330747; doi:10.1186/s12968-018-0516-1)

**
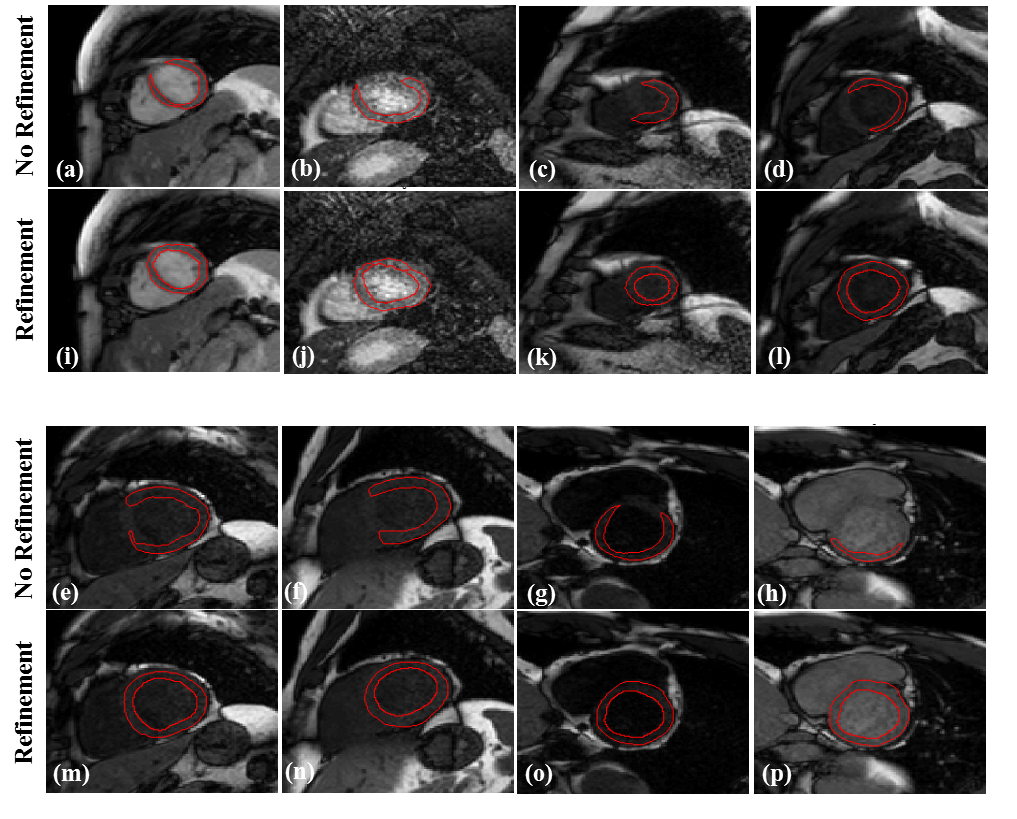
**

**Figure S2.** Example results of the automatic segmentation before and after refinement.

Supplement: Supplementary file 2 — Figure S2. Example results of the automatic segmentation before and after refinement. (DOCX 470 kb) [file 12968_2018_516_MOESM2_ESM.docx]
