# Supplementary material for: Automated analysis of cardiovascular magnetic resonance myocardial native T1 mapping images using fully convolutional neural networks
Source: J Cardiovasc Magn Reson. 2019 Jan 14;21:7. doi: 10.1186/s12968-018-0516-1 (PMC6330747; doi:10.1186/s12968-018-0516-1)
Supplement: Supplementary file 3 — Figure S3. Effect of area filter on the output of the neural network. (a) input T1 weighted image; (b,c) network output before and after area filtering, respectively; (d) manual segmentation. (DOCX 225 kb) [file 12968_2018_516_MOESM3_ESM.docx]

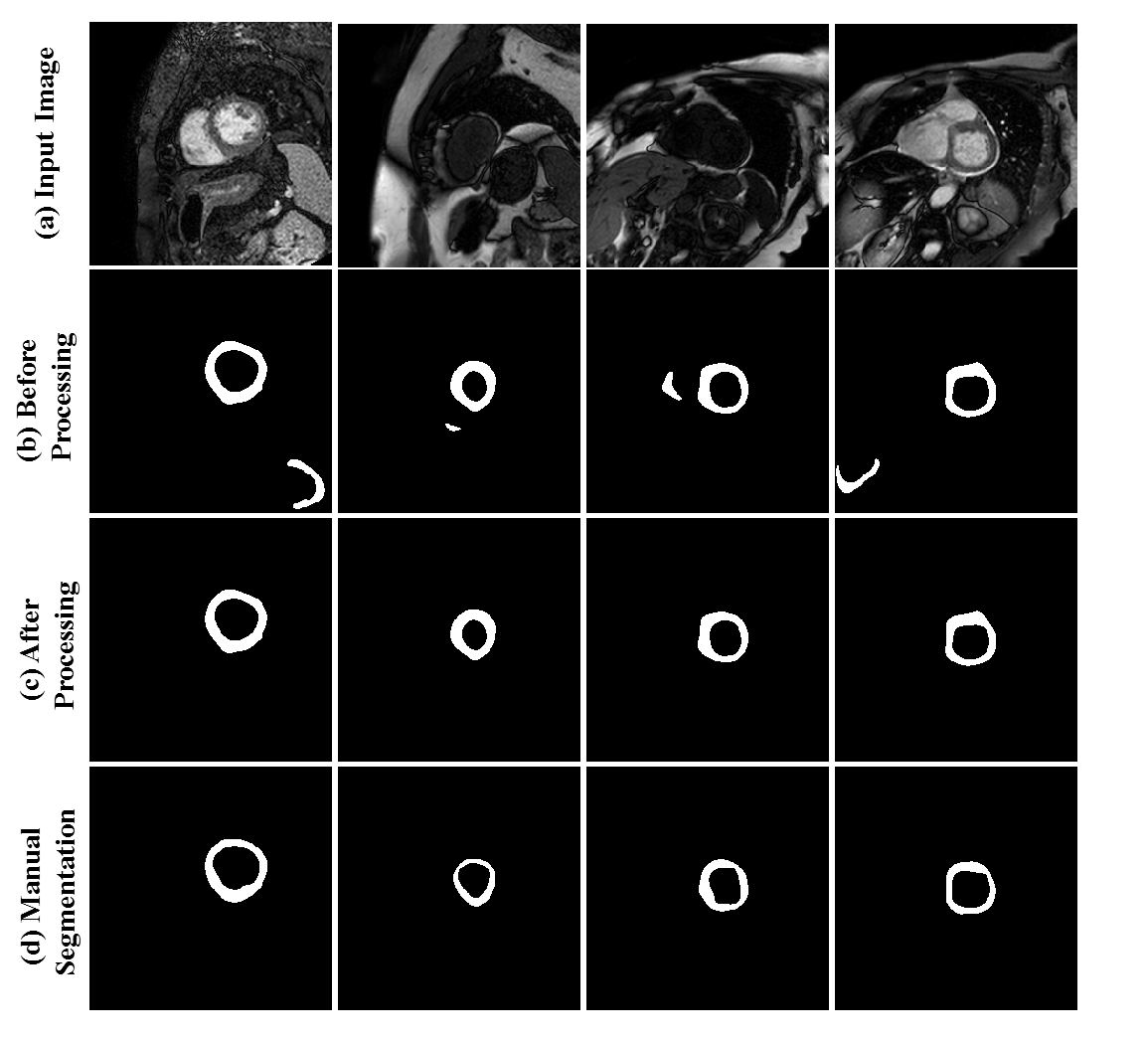


**Figure S.3** Effect of area filter on the output of the neural network. (a) input T1 weighted image; (b,c) network output before and after area filtering, respectively; (d) manual segmentation.
